# Model-based vascular elastography improves the detection of flow-induced carotid artery remodeling in mice

**DOI:** 10.1038/s41598-017-12321-7

**Published:** 2017-09-21

**Authors:** Vyacheslav A. Korshunov, Hexuan Wang, Rifat Ahmed, Deanne M. Mickelsen, Qian Zhou, Chen Yan, Marvin M. Doyley

**Affiliations:** 1 Aab Cardiovascular Research Institute, Department of Medicine, University of Rochester School of Medicine and Dentistry, Rochester, NY USA; 2 Department of Biomedical Genetics, University of Rochester School of Medicine and Dentistry, Rochester, NY USA; 3 Department of Electrical & Computer Engineering, University of Rochester and Hajim School of Engineering & Applied Sciences, Rochester, NY USA

## Abstract

Increased arterial thickness measured with ultrasound correlates with future cardiovascular events, but conventional ultrasound imaging techniques cannot distinguish between intima, media, or atherosclerotic plaque in the carotid artery. In this work, we evaluated how well vascular elastography can detect intimal changes in a mouse model of carotid remodeling. We ligated the left external and internal branches of the carotid artery of male FVB mice and performed sham operations for 2 weeks. High-resolution ultrasound imaging accurately detected lower blood velocities and low blood volume flow in the carotid arteries after ligation in FVB mice. However, ultrasound could not detect differences in the carotid wall even at 2 weeks post-surgery. The Young’s modulus was measured based on displacements of the carotid artery wall, and Young’s modulus was 2-fold greater in shams at 1 week post ligation, and 3-fold greater 2 weeks after ligation. Finally, the higher Young’s modulus was most associated with higher intimal thickness but not medial or adventitial thickness as measured by histology. In conclusion, we developed a robust ultrasound-based elastography method for early detection of intimal changes in small animals.

## Introduction

Diagnostic ultrasound can assess the progression of vascular disease in patients^1^, but further improvements are needed. Carotid intima-media thickness (IMT) is a surrogate measure of atherosclerosis, and increased IMT correlates with future cardiovascular events^2^. Although carotid IMT measurements improve the Framingham risk score^3^, there are concerns about the large variances^4^ and poor reproducibility of IMT values obtained from the same individuals^5^ — a problem caused by the poor out-of-plane (elevational) resolution of one-dimensional (1D) transducer arrays^4^ used in IMT assessment. Additionally, it is difficult to detect preclinical atherosclerosis or classify different plaque components^6^ with standard ultrasound. This limitation occurs because – except for calcification – ultrasound backscatter signal strength is independent of plaque composition. Emerging ultrasound techniques such elastography show promise in overcoming these limitations by visualizing changes in the biomechanical properties (stiffness) in the underlying tissues’ different echogenicities.

Vascular elastography^7–9^ should allow clinicians to detect early structural changes in carotid arteries. Elastography was originally developed in the late 1980s^10^ to improve the differential diagnosis of breast cancer. The success of elastography in breast cancer imaging^11,12^ has inspired researchers to explore different clinical applications including prostate imaging^13,14^, cardiovascular imaging^15–18^, and guiding minimally invasive therapeutic techniques^19–21^. The general principle of elastography can be summarized as follows: (1) use either a quasi-static, harmonic, or transient mechanical source to perturb the tissue; (2) use ultrasound to measure the resulting tissue motion; and (3) use either a simple or inverse recovery method to infer the biomechanical properties of the underlying tissue. Over the last decade, we and others have developed both minimally invasive^7,8,22^ and non-invasive^9,15,17,23,24^ elastographic methods for visualizing the Young’s modulus distribution (stiffness maps) of vascular tissues. Most research in vascular elastography has been focused on improving the detection of rupture-prone atherosclerotic plaques^15,25,26^. Consequently, evaluation of the technique has been performed in either large animals^27^ or in trials using phantom arteries^9^. The long-term objective of this work is to establish arterial stiffness as an imaging biomarker for detecting preclinical arthrosclerosis in the carotid artery.

The goal of this research is to corroborate our hypothesis that vascular elastography can detect early structural changes in the arterial tissues. The motivation for conducting this study is that thickening of the intima occurs in the early stages of atherosclerotic process; therefore, an imaging system that can detect these early changes would allow clinical researchers to design interventions that can be evaluated in smaller samples and require shorter follow-up to assess effectivity. Our group has developed a mouse model of low flow-induced carotid thickening without atheroma^28^, which we will use to study how well vascular elastography can assess structural changes in carotid arteries.

## Results

### Hemodynamic changes in the carotid artery after ligation in FVB mice

**Figure 1:**
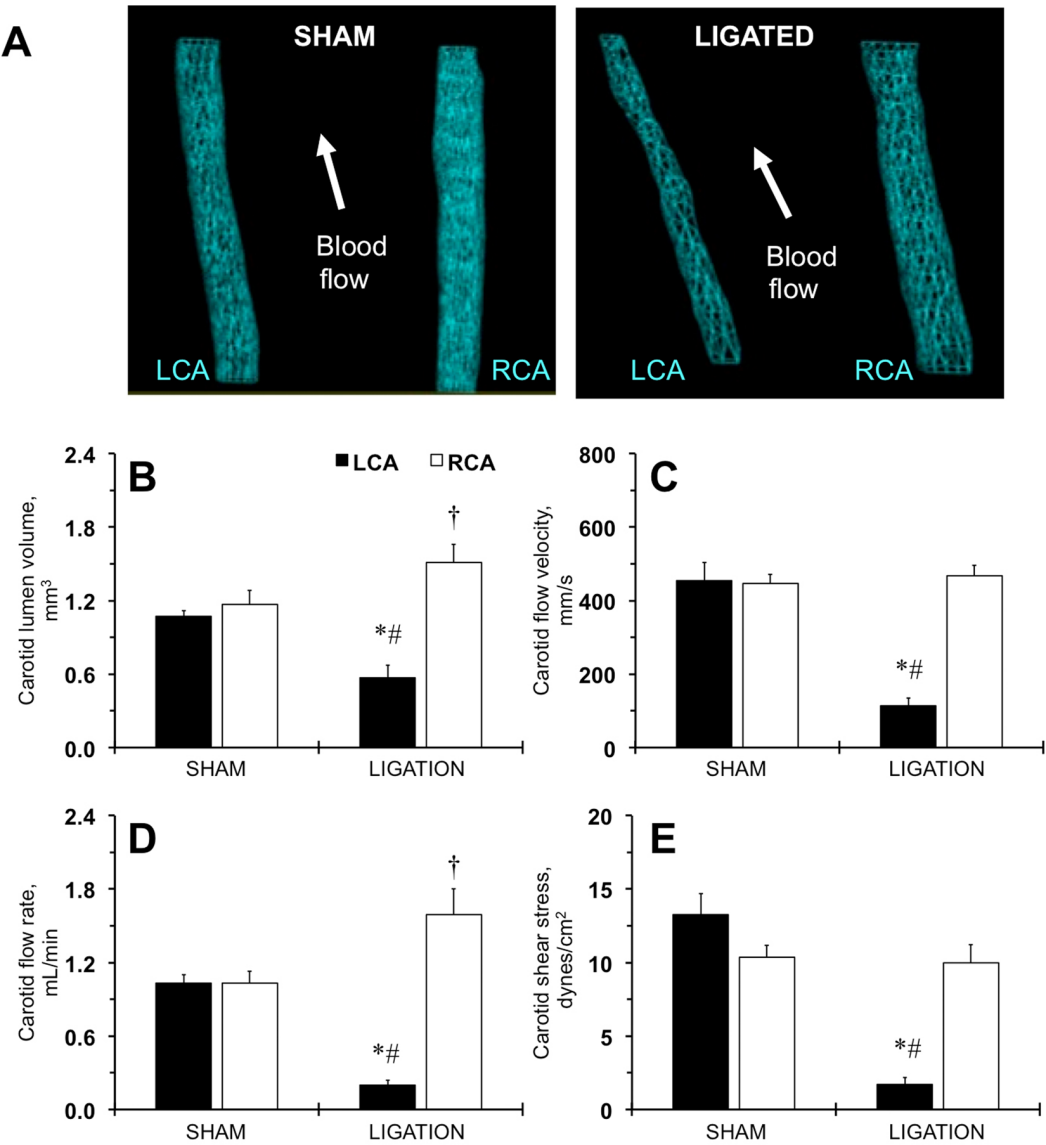
Hemodynamic changes in carotid arteries determined by ultrasound in FVB mice two weeks after ligation. (**A**) Representative 3D-images of the lumen of the sham-operated (SHAM) or ligated (LIGATED) mice. LCA, left carotid artery. RCA, right carotid artery. Arrows show direction of the blood flow. (**B**) Carotid lumen volume, mm^3^. (**C**) Carotid blood flow velocity rate, mm/s. (**D**) Carotid blood flow volume rate, mL/min. (**E**) Carotid shear stress, dynes/cm^2^. Black bars are LCA. Open bars – RCA. SHAM and LIGATED mice are shown on X-axis. Values are mean ± SEM. *p < 0.05 vs. LCA, SHAM. ^†^p < 0.05 vs. RCA, SHAM. ^#^p < 0.05 vs. RCA, LIGATED. n = 5–6 per group.

Our investigation of several inbred mouse strains suggested that FVB and SJL/J mice developed the most robust carotid intima and media thickening in response to low blood flow^29,30^. We previously reported that FVB mice have increased maximal constriction of aortic rings as a response to vasoconstrictors (potassium chloride or phenylephrine) compared to other inbred strains of mice^31^. Our Vevo LAB analyses found no changes in heart functions in shams and ligated FVB mice 1 week or 2 weeks after the procedure (Table S1; Table 1). Diastolic and systolic lumen diameters of the ligated left carotid artery (LCA) were similar or even greater in size compared with shams, but lower than in contralateral right carotid artery (RCA) over the 2-week time period (Table 1). 3D imaging of the LCA after ligation showed significant reduction of the carotid lumen volume (Fig. S1B; Fig. 1B). Blood flow velocity and its integrated parameters were also significantly reduced in ligated LCA (Fig. S1C; Fig. 1C; Table 1). Compensation in the contralateral artery (RCA) resulted in transient increase of the carotid diameters and more consistent lumen volume increase after ligation (Table 1; Fig. S1B; Fig. 1B). However, RCA blood velocity parameters did not reach statistical significance vs. shams at both time points (Fig. S1C; Fig. 1C; Table 1). As we reported previously^28,29^, the volume blood flow rate decreased by >80% in the ligated LCA and increased by ~60% in the contralateral RCA after 2 weeks in mice (Fig. 1D). The relative RCA volume flow seems to be a little higher at 1 week after ligation (Fig. S1D). The calculated shear stress was consistently reduced in the ligated LCA but normalized in the RCA after ligation (Fig. 1E; Fig. S1E). Thus, ultrasound assessment accurately determined hemodynamic profiles in the carotid arteries after ligation in FVB mice.

**Table 1:** Ligation procedure significantly changed blood flow profiles of carotid arteries in FVB mice over a 2 week time period.

Parameter Group	1 week post-surgery		2 weeks post-surgery	
	Sham, n = 5	Ligated, n = 5	Sham, n = 5	Ligated, n = 6
**Left Carotid Artery**				
Doppler heart rate, beats/min	517 ± 6	514 ± 25	541 ± 11	537 ± 6
Diastolic diameter, mm	0.36 ± 0.02	0.48 ± 0.03^*^	0.38 ± 0.01	0.40 ± 0.04^†#$^
Systolic diameter, mm	0.30 ± 0.01	0.45 ± 0.03^*^	0.30 ± 0.00	0.38 ± 0.04^†$^
End diastolic velocity, mm/s	74 ± 16	1 ± 0^*‡^	118 ± 12	2 ± 1^†#^
Peak systolic velocity, mm/s	597 ± 49	191 ± 30^*‡^	828 ± 89^*^	216 ± 44^†#^
Mean gradient, mmHg	0.42 ± 0.08	0.05 ± 0.0 1^‡^	0.87 ± 0.18^*^	0.06 ± 0.03^†#^
Velocity time integral, mm	20.6 ± 3.0	2.8 ± 0.3^*‡^	28.5 ± 2.7	4.1 ± 0.8^†#^
***Right Carotid Artery***				
Doppler heart rate, beats/min	511 ± 11	528 ± 16	546 ± 8	540 ± 8
Diastolic diameter, mm	0.39 ± 0.02	0.51 ± 0.01^*^	0.43 ± 0.01	0.49 ± 0.03
Systolic diameter, mm	0.30 ± 0.02	0.40 ± 0.01^*^	0.33 ± 0.01	0.39 ± 0.03
End diastolic velocity, mm/s	95 ± 22	108 ± 16	90 ± 12	86 ± 8
Peak systolic velocity, mm/s	807 ± 62	919 ± 135	850 ± 51	887 ± 59
Mean gradient, mmHg	0.78 ± 0.13	1.03 ± 0.27	0.81 ± 0.10	0.89 ± 0.10
Velocity time integral, mm	26.3 ± 2.6	27.6 ± 2.9	24.2 ± 1.6	26.5 ± 1.3

Parameters are shown as mean ± SEM. *p < 0.05 vs. Sham, 1 week. ^†^p < 0.05 vs. Sham, 2 weeks. ^‡^p < 0.05 vs. Ligated RCA, 1 week. ^#^p < 0.05 vs. Ligated RCA, 2 weeks. ^$^p < 0.05 vs. Ligated LCA, 1 week. n, Number per group.

### Evaluation of the carotid remodeling in FVB mice by ultrasound

**Figure 2:**
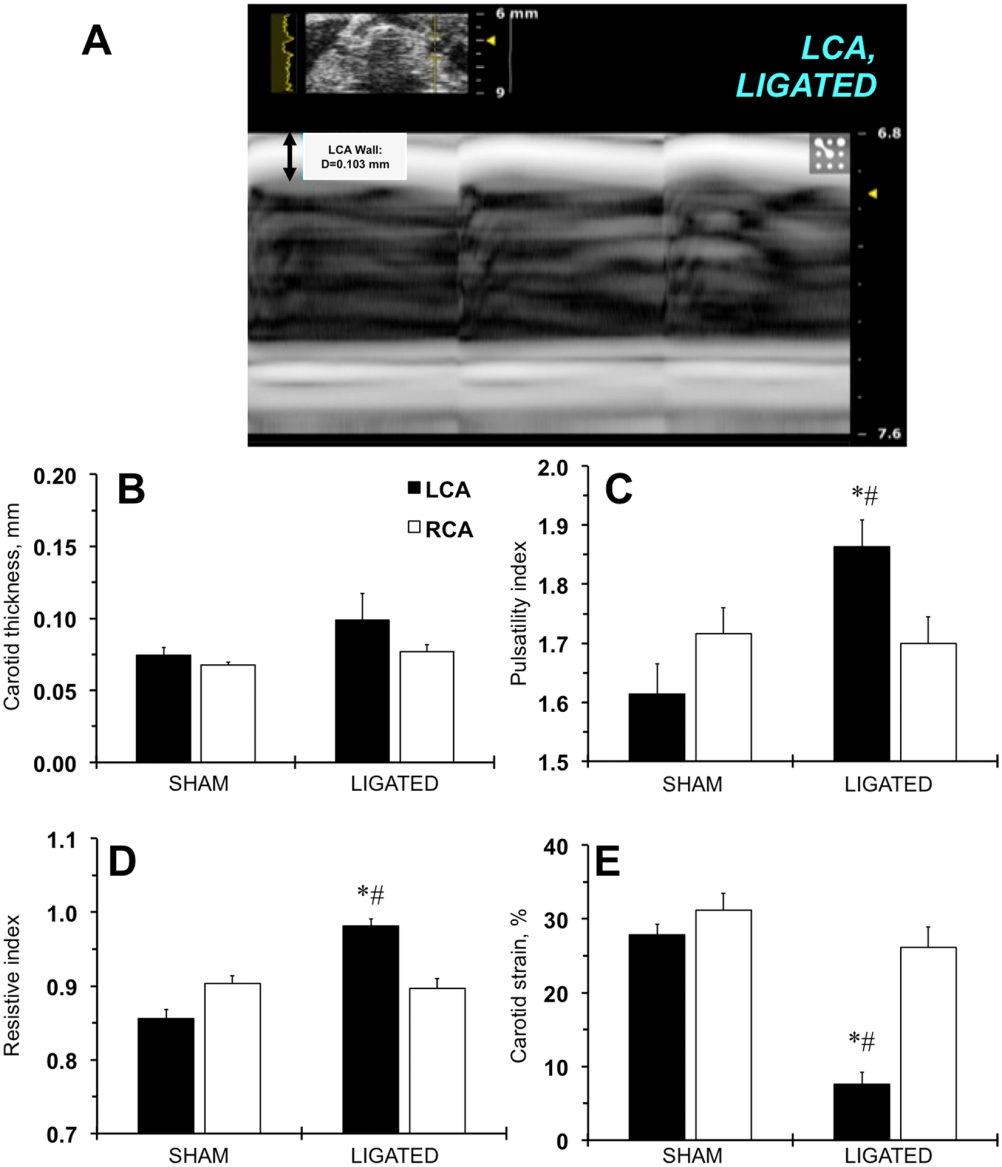
Ultrasound profiles of the carotid artery wall in FVB mice two weeks after ligation. (**A**) A representative image of the ligated carotid artery in an ultrasonography M-mode. A bidirectional arrow and a number define carotid artery wall thickness. LCA, left carotid artery. (**B**) Carotid artery wall thickness, mm. (**C**) Carotid artery pulsatility index. (**D**) Carotid artery resistive index. (**E**) Carotid artery strain, %. Black bars – left carotid artery (LCA). Open bars – right carotid artery (RCA). SHAM and LIGATED mice are shown on X-axis. Values are mean ± SEM. *p < 0.05 vs. LCA, SHAM. ^#^p < 0.05 vs. RCA, LIGATED. n = 5–6 per group.

One of the major limitations of ultrasound imaging is the low resolution of artery wall changes that can detect atheromas in mice^32^. Our previous histological analyses of changes over the 4 week time period showed a detectible increase in carotid remodeling in FVB mice between 1 and 2 weeks after ligation^30^. Ultrasound was not able to accurately measure carotid thickness in LCA 1 week after ligation (not shown). Even at 2 weeks post-surgery, the changes in ligated LCA carotid thickness did not reach statistical significance (Fig. 2A,B). Despite these limitations, we found that the pulsatility and resistive indexes increased in ligated LCA after 1 and 2 weeks using the Vevo LAB package (Fig. S2A,B; Fig. 2C,D). Finally, the calculated carotid strain decreased significantly in ligated LCA at both time points (Fig. S2C; Fig. 2E). There was a small reduction in carotid strain in the sham LCA at 1 week after the surgery (Fig. S2C). Our results confirmed the limited ability of ultrasound in detecting early changes in carotid thickness in a mouse.

### Elastographic signature of the carotid arteries in FVB mice after ligation

**Figure 3:**
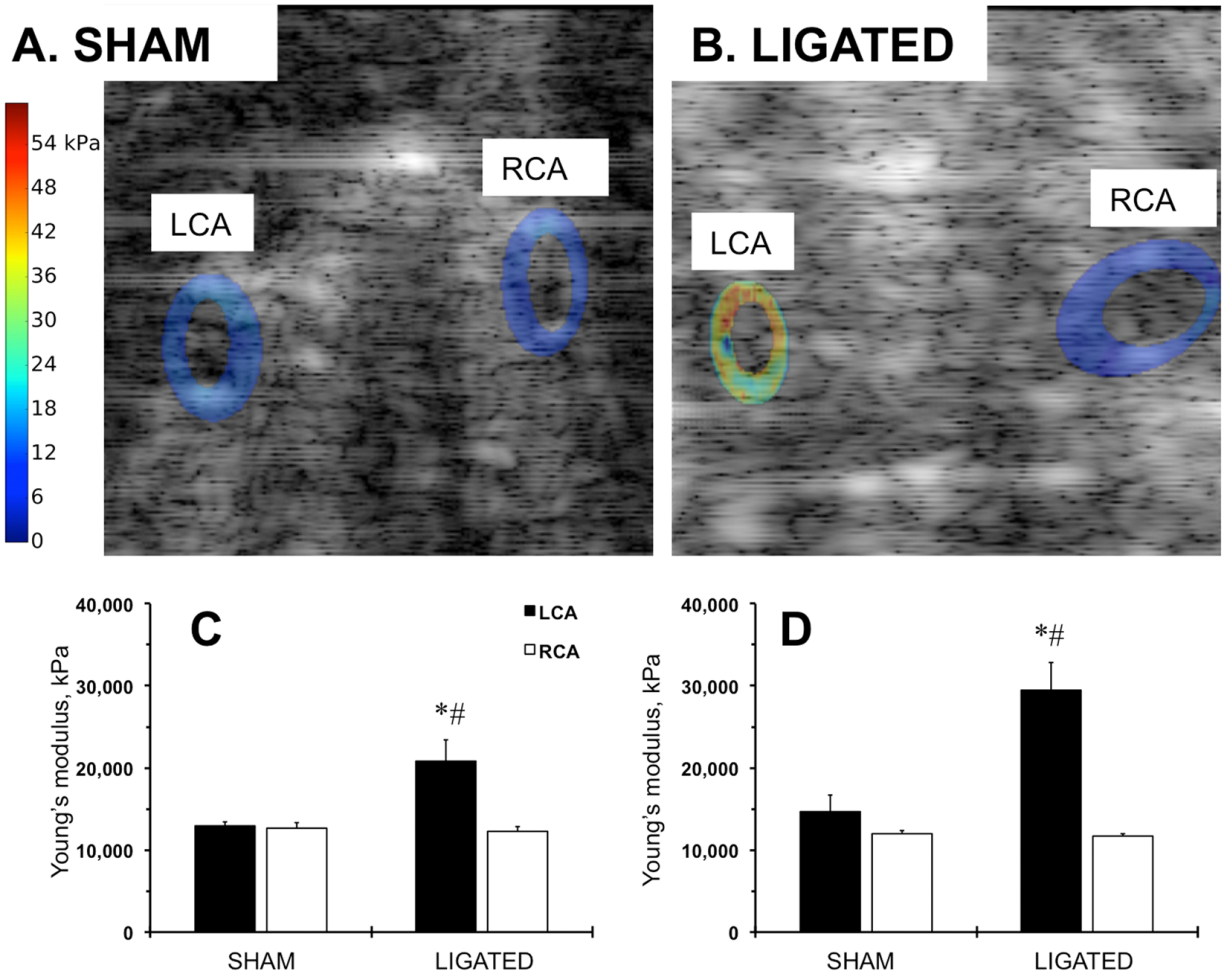
Elastography profiles of the carotid arteries in FVB mice after ligation. (**A**) Computed sonograms of the carotid arteries at 2 weeks after sham operation. (**B**) Computed sonograms of the carotid arteries at 2 weeks after ligation. LCA, left carotid artery. RCA, right carotid artery. The Young’s modulus is color-coded: lowest (zero) is set in blue; highest value (54 kPa) is in red. (**C**) Quantitative analysis of the Young’s modulus at 1 week post-surgery. (**D**) Quantitative analysis of the Young’s modulus at 2 weeks post-surgery. Black bars – LCA. Open bars – RCA. SHAM and LIGATED mice are shown on X-axis. Values are mean ± SEM. *p < 0.05 vs. LCA, SHAM. ^#^p < 0.05 vs. RCA, LIGATED. n = 5 per group.

We then computed Young’s modulus by applying our iterative inversion technique^33^ to the ultrasonically measured displacements of the carotids. We observed that there were differences in Young’s modulus in the LCA and RCA 2 weeks after the ligation and sham operations (Fig. S3A,C; Fig. 3A). However, the low blood flow increased the Young’s modulus of the ligated LCA during that time period (Fig. S3B,D; Fig. 3B). Additionally, elastography allowed us to determine when the remodeling extended into the ligated carotids and showed non-uniform distribution of Young’s modulus compared with shams or contralateral RCA (Fig. S3; Fig. 3B). Quantitative analyses confirmed a gradual increase (from 2-fold to 3-fold) in the ligated LCA Young’s modulus 2 weeks after the surgery (Fig. 3C,D). These findings suggest that elastography techniques enable detection of increased thickness of the carotid artery in a mouse.

### Histological analyses of carotid remodeling in FVB mice

**Figure 4:**
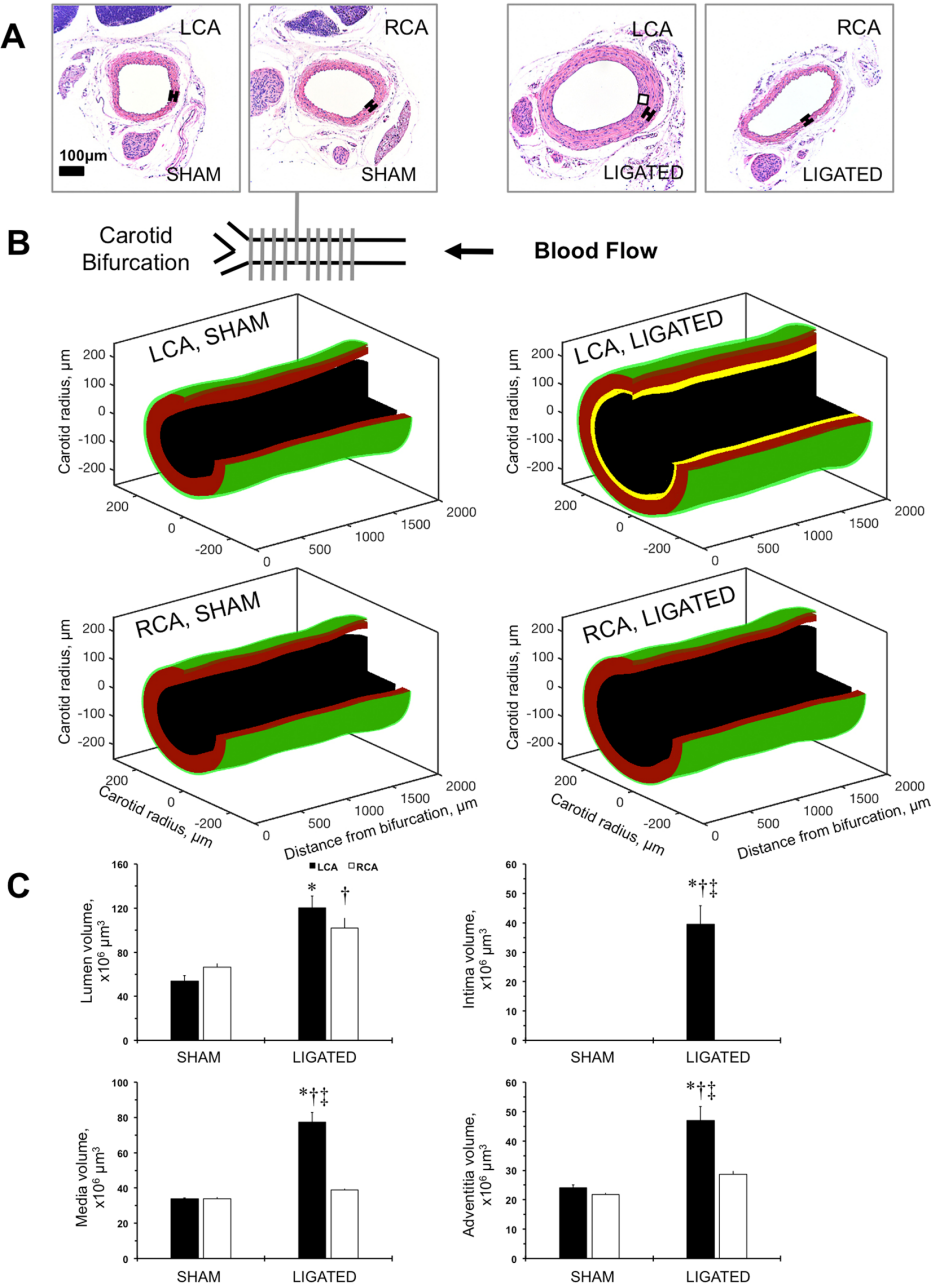
Reconstruction of the carotid remodeling in FVB mice after ligation. (**A**) Representative cross-sections of the left carotid artery (LCA) and right carotid artery (RCA) from sham-operated (SHAM) or ligated (LIGATED) mice. Magnification is 20x. Bar is 100 *μ*m. (**B**) A drawing showing carotid artery at the bifurcation point. Series of cross-sections marked with grey perpendicular lines (every 200 *μ*m) are covering 2,000 *μ*m carotid artery length. Arrow shows direction of blood flow. 3D-reconstruction of the carotid artery compartments based on series of histological sectioning. It was not possible to measure the intimal layer in non-ligated arteries. Carotid compartments are color-coded: black is lumen area, yellow is intimal area, red is medial area, and green is adventitial area. (**C)** Carotid artery component volumes: lumen, intima, media, and adventitia. Black bars - LCA. Open bars - RCA. SHAM and LIGATED mice are shown on X-axis. Values are mean ± SEM. *p < 0.05 vs. LCA, SHAM; ^†^p < 0.05 vs. RCA, SHAM. ^‡^p < 0.05 vs. RCA, LIGATED. n = 5–6.

To validate ultrasound-based analyses, we performed serial sectioning of the carotid artery from the carotid bifurcation to the proximal part of the carotid artery as we described previously^28,34^. The ligation procedure significantly increased carotid thickness in the LCA of FVB mice (Fig. 4A). We confirmed the uniform wall thickening of the ligated LCA in 3D after ligation based on morphometric analyses of the multiple cross-sections (Fig. 4B; Fig. S4). As we previously found in FVB mice^29,30^, the carotid compartmental volumes quantitatively reflected the 3D distribution of the carotid remodeling (Fig. 4C). Therefore, the histological analyses revealed significant carotid thickening without decline in lumen in mice after ligation.

### Relationship between ultrasound and histological methods of the carotid remodeling in FVB mice

**Figure 5:**
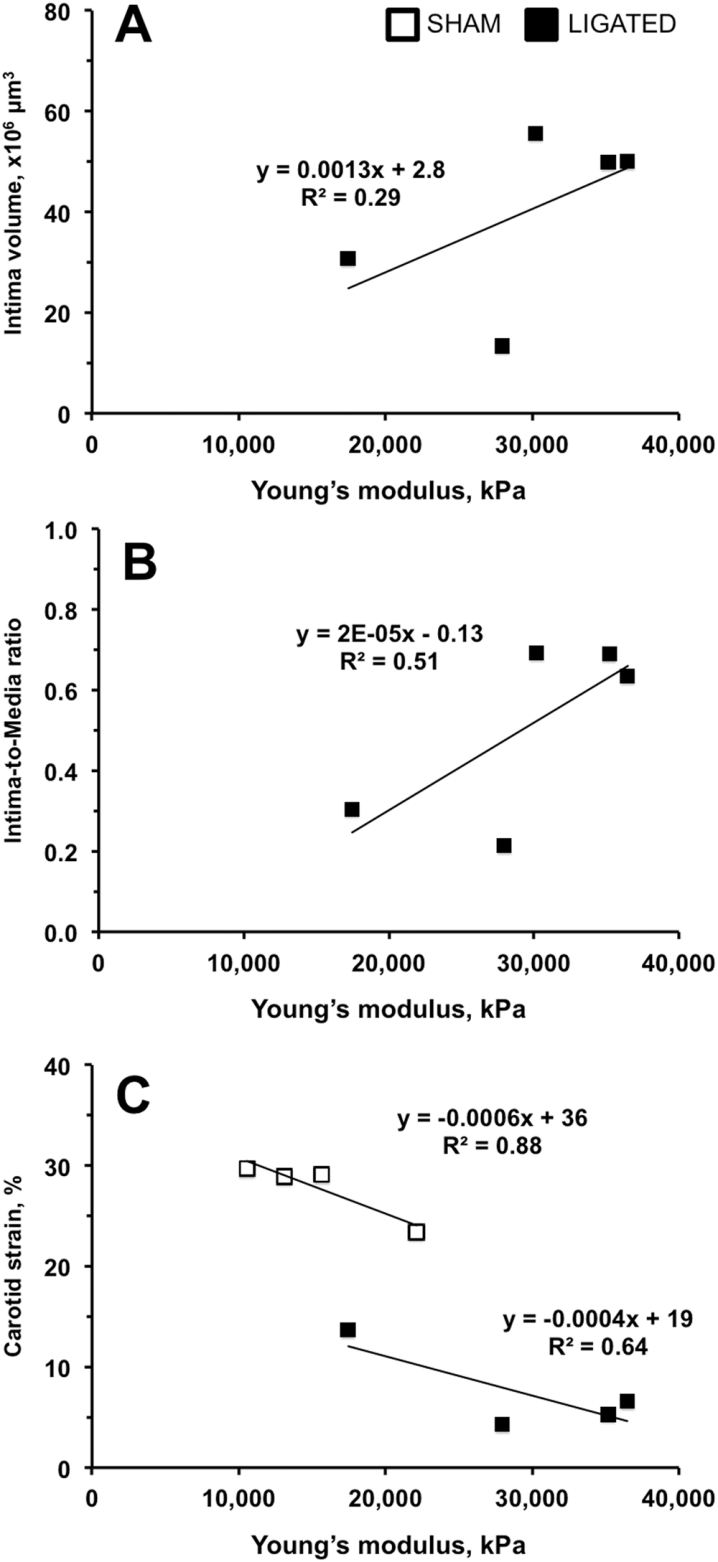
Relationships between the Young’s modulus and the left carotid remodeling in FVB mice after ligation. Open squares are sham-operated animals (SHAM). Black squares are ligated mice (LIGATED). Intima volume (**A**) and Intima/Media Ratio (**B**) positively correlate with the Young’s modulus. It was not possible to measure the intimal layer in non-ligated arteries. (**C**) The carotid strain negatively correlated with the Young’s modulus. n = 5.

In order to validate histological changes in the carotid arteries, we correlated the elastography profiles in the same animal at the 2-week time-point (Fig. 5). We observed a strong correlation between arterial intimal volume and Young’s modulus, especially when this was normalized to the media in the ligated LCA (Fig. 5A,B). In contrast, medial and adventitial volumes negatively correlated with the Young’s modulus after ligation, while shams exhibited no correlation (Fig. S5). Similarly, reduction in carotid strain was related to a dramatic increase in the Young’s modulus after ligation (Fig. 5C). These results suggest that ultrasound elastography could potentially be used to predict pathological remodeling.

## Discussion

A major finding of our study is that ultrasound-based elastography enabled early detection of increase in intima in a mouse model of flow-induced vascular remodeling. Significantly higher Young’s modulus values corresponded to histological changes in the mouse carotid intimal compartment. This is in contrast to standard ultrasound imaging that gives poor measurements of carotid artery wall changes in mice. Moreover, intimal thickness determined by 3D-projections of carotid histology correlated the most with the higher Young’s modulus. Finally, we found that the standard ultrasound technique is accurate in determining hemodynamic profiles in the carotid arteries after ligation. To our knowledge, this work is one of the first studies to use model-based vascular elastography to investigate carotid artery remodeling in a mouse.

The majority of clinical and experimental efforts focused on artery wall disease use a surrogate parameter for atherosclerosis, the carotid IMT, which correlates with cardiovascular events^2^. Among mechanical forces that affect the vascular wall, shear stress has been the most studied^35^. A very popular animal model to mimic carotid remodeling in patients was developed by our group using a ligation procedure on the mouse carotid artery branches^28^. Our partial ligation resulted in comparable increase in intima vs. the complete cessation of blood flow in the mouse carotid^36^. Low blood flow and low shear stress (~90%) are the primary hemodynamic forces that induce vascular remodeling in this model, as was shown in an early modification of the ligation procedure on the major branches of the common carotid artery^37^. Remodeling response requires a threshold below 50% of the original blood flow, as ligation of one branch of the carotid showed no remodeling in a mouse^38^. There were no changes in systolic blood pressure or heart rate during the 4-week time period after the carotid ligation procedure in mice^28,30^. In the current study, we confirmed that heart functions remain unaffected two weeks after surgery. The latter suggests that the pathologic blood flow profile is uniquely localized to the ligated LCA in this mouse model. Blood flow velocity profiles were accurately assessed with ultrasound and the calculated volume blood flow was similar to values we previously measured by direct application of the transonic flow-probe^28,30^. The obvious advantage of ultrasound is its ability to trace the natural history of the carotid thickness without the mechanical damage associated with invasive blood flow measurements^28^.

Previous experiments in ApoE^−/−^ mice showed validity in assessing carotid atherosclerosis using ultrasound^32^. In particular, the authors reported that low total longitudinal displacement of the common carotid artery was strongly associated with atherosclerotic plaque development in ApoE^−/−^ mice, similar to humans. Similarly, carotid strain was significantly reduced upon ligation in our study, which occurred because the procedure increased the Young’s modulus (stiffness) of the arterial wall. Atherosclerotic progression in coronary arteries developed almost exclusively in areas of low shear stress in humans^39,40^. These findings were verified experimentally in ApoE^−/−^ mice that were challenged with the partial ligation model^41^. Carotid atherosclerotic plaque formation was accelerated, and it peaked 2 weeks after surgery. The authors were able to detect early changes in blood flow profiles in the carotid (1-week time-point) using ultrasound in this model^41^. Researchers have used high-frequency ultrasound to measure arterial surface roughness, a useful parameter for detecting early atherosclerosis in the carotid arteries in ApoE^−/−^ mice^42^, but none of the imaging modalities have sufficient resolution to visualize the earliest stages of the atherosclerotic process. Therefore, histological evaluation of the carotid arteries is the standard technique used to assess the degree of pathological carotid remodeling in animals.

Blood flow cessation in the carotid artery can affect the distribution of the intima over the vessel length^36^. In the current study, we used a histological approach to confirm uniform carotid wall thickening with serial sectioning of 2 mm lengths of the carotid artery as before^28^. In addition, we visualized histological changes in 3D-projection after ligation by applying the recently published method we developed for vein graft formation in mice^34^. The volume of the ligated LCA lumen measured with ultrasound was lower than that measured with histology. Specifically, the average length of the carotid proximal to bifurcation measured with ultrasound was 4 mm, compared to 2 mm measured with 3D histology for the same vessel. We believe that greater tissue shrinkage of the carotids from the sham-operated mice due to fixation was the main source of such difference^43^. Specifically, sham LCA lumen diameter measured using histology was 0.23 mm, while systolic lumen diameter was 0.30 mm vs. diastolic diameter at 0.38 mm. Hence, ligated LCA lumen diameter was 0.32 mm using histology, while systolic lumen diameter was 0.38 mm vs. diastolic diameter at 0.40 mm. Thus, ultrasound imaging is limited compared with histological evaluation of the carotid artery wall remodeling, but ultrasound provides robust blood flow profiles and lumen validation for arterial changes in mice.

Vascular elastography is an emerging ultrasound method for visualizing the biomechanical properties of vascular tissues. It measures strain or displacements induced in arteries in response to the pulsating blood pressure^7–9^, information that when combined with continuum mechanical modeling can produce spatial maps of the intrinsic biomechanical properties^44–46^. Several groups, including ours, are investigating the feasibility of using elastography to improve the detection of rupture-prone atherosclerotic plaques^15,25,26^. More recently, we demonstrated that vascular elastography can visualize principal stresses within vascular tissues, the primary factor that governs the mechanical stabilities of plaques^22^. We have also applied vascular elastography to phantoms, and to carotid arteries from rabbits and humans^9^. Despite the encouraging results for large-size arteries, very little work has been done with small animals. The mouse elastography revealed that Young’s modulus increased noticeably in the LCA after ligation only. More specifically, it was 2-fold greater than the Young’s modulus measured in shams 1 week post-ligation, and 3-fold greater 2 weeks post-ligation. Other physiological parameters (pulsatility and resistive indexes) measured in the ligated LCA increased marginally (~10%) at each time-point. We expected an increased Young’s modulus in remodeled carotids of FVB mice because this strain of mice had greater vasoreactivity to vasoconstrictors than other inbred mouse strains that are prone to increase in intima after vascular injury^31,47^. Our study suggests that genetic predisposition to higher contractility in FVB mice can be detected by elastography as one of the key biological processes (inflammation, cell apoptosis, calcification, etc.) during intimal proliferation. Future focus on biomechanical changes in mice might uncover important signaling pathways that regulate early pathophysiological changes in the carotid artery.

For over two decades, the intima has been proposed as the soil of atherosclerosis and restenosis, and the primary pathogenic compartment in vascular remodeling^48^. Like atherosclerotic plaques, histologically-assessed intimal volume positively correlated with Young’s modulus values in the ligated LCA. Interestingly, medial or adventitial thickening in the ligated LCA had no relationship with Young’s modulus. Vascular elastography is a new imaging modality that is constantly being improved. For example, the current approach is performed using strain information obtained from a single cross-section of the artery, which could limit the accuracy of the elastogram. The biomechanics of the vessels is a complex three-dimensional problem; more mechanical information (strains) is needed to give precise estimates of Young’s modulus. We expect 2D ultrasound matrix arrays will address the problem when they become more readily available. Since Young’s modulus depends on both pressure (boundary conditions) and the mechanical properties, we plan to conduct further investigation to determine which of these two variables is most responsible for the observed changes. The results of this study will be the subject of a future communication in this journal. In the clinic, vascular elastography of the carotid artery is performed with lower frequency ultrasound (7–12 MHz) than used in this study, which could lower the sensitivity of vascular elastography.

Despite these limitations, the current approach using vascular elastography should prove useful in comparative studies where the absolute value of Young’s modulus is not important. Furthermore, it should allow high throughput analyses of pathological vascular remodeling in genetically manipulated or pharmacologically treated mice. We were able to detect an unsuccessful ligation procedure using ultrasound imaging in one animal at 1 week post-surgery (not shown). Later, the Young’s modulus and histological measurements in this animal were found to be similar to those in sham-operated mice (not shown). Our new experimental approach could potentially be translated to human trials. Elastographic detection of the stiffer intimal compartment could become a reliable measurement of initiation and progression of atherosclerosis. Previously, pharmacologic interventions in a small group of Mexican patients (VYCTOR study) showed not only a decrease in carotid thickness, but also a decrease in carotid stiffness^49^.

## Methods

### Animals

Experiments were performed on male FVB/NJ (FVB) mice at age 6–8 weeks purchased from the Jackson Laboratory (Bar Harbor, ME, USA). Mice were housed individually at a 12 hours light and 12 hours dark cycle (lights on from 6 a.m. to 6 p.m.) with free access to chow and water. The University of Rochester Animal Care Committee approved all procedures on animals; the procedures were conducted within the guidelines of the National Institutes of Health for use of laboratory animals.

### Carotid artery ligation

FVB mice (n = 5–6 per group) underwent ligation of the left external and internal branches of the carotid artery with 6–0 silk as described^28^. The branches of the left carotid artery were isolated but not ligated in sham-operated mice. Mice were anesthetized with a cocktail of ketamine and xylazine (130 mg/kg and 9 mg/kg, respectively, i.p.). Animals were also injected with analgesic Flunixin meglumine (120 mg/kg, i.p.) immediately before and within 12–24 hours after the surgery. Mice were housed individually in an enhanced environment (a plastic house and bedding material) under pathogen-free conditions for 2 weeks post-surgery.

### Ultrasound measurements of the carotid arteries and heart

We used a high-resolution ultrasound system (Vevo2100, FUJIFILM VisualSonics; Toronto Canada) to evaluate carotid remodeling and heart functions in mice, as we recently reported after vein graft surgery^34^. Mice were anesthetized with isoflurane (gas flow at 100–200 mL/min and vaporizer at 2–3%) and monitored to maintain heart rate above 500 beats/min during measurements. We assessed heart functions, hemodynamic changes, and vascular wall remodeling at 1 week and 2 weeks post-surgery. All imaging was performed with a 128-element transducer operating at 40 MHz (MS550D). Cardiac functions calculated from M-mode of the short axis view in sham-operated and ligated mice were analyzed at 1 and 2 weeks after surgery. A 3-dimentional (3D) image was captured by the Vevo2100 automated stage with 500 *μ*m-step increments just caudal to the external/internal carotid bifurcation acquired using power 3D Doppler in a cross sectional view. We calculated lumen volume (over a 4 mm length), and analyzed blood flow rates, carotid artery wall thickness, and stiffness using Vevo LAB analysis software. Carotid blood flow volume rate was calculated based on the following formula: blood flow = (heart rate × lumen area × velocity time integral)/1,000. Estimation of the mean shear stress was calculated based on the lumen radius and volume flow rate as previously described^28^. Carotid artery stiffness was determined by the following calculation: resistive index = (peak systolic velocity - end diastolic velocity)/peak systolic velocity; pulsatility index = (peak systolic velocity - end diastolic velocity)/velocity time integral of mean velocity; carotid strain = ((systolic lumen diameter - diastolic lumen diameter)/systolic lumen diameter) ×100. This assumption is valid only for cases where there is parabolic flow. For other cases, we could image through a stand-off layer of known modulus, and reconstruct modulus elastograms using displacement boundary conditions. Since this reconstruction approach provides relative stiffness estimates^50^, we will use knowledge of the mechanical properties in the stand-off layer to calibrate modulus elastograms.

### Elastography using radio frequency (RF) ultrasound

We applied our vascular elastography image formation process^51^ to RF echo data collected from FVB mice with the Vevo2100 system. Specifically, we used a two-step process to visualize the Young’s modulus (stiffness) distribution within the vessels. First, we computed axial and lateral displacements by applying a two-dimensional echo tracking technique described in^15^ to the acquired RF echo frames. All echo tracking was performed with 100 *μ*m by 25.4 *μ*m kernels that overlapped by 80% in both the axial and lateral dimensions. Second, we applied our iterative inversion technique to ultrasonically measured tissue displacements. In these studies, we acquired echo frames from the transverse planes of the carotid artery. We assumed a uniform pressure of 131 mmHg was exerted on the inner lumen. The systolic blood pressure values were obtained using the tail-cuff method in our previously published studies on FVB mice^30,52^. There were no changes in systolic blood pressure during sham and ligation surgery over the 4-week time course in FVB mice. Our experiments in FVB mice are in agreement with early experiments in rabbits that show no change in blood pressure after ligation of the carotid artery^53^. In addition, our group recently reported strong correlation between the indirect tail-cuff plethysmography and direct radiotelemetry methods of blood pressure measurements in mice^54^. We are currently limited in our techniques to accurately measure local arterial pressure profiles upon carotid ligation in a mouse carotid artery. Therefore, absolute Young’s modulus values may not represent arterial stiffness changes during each cardiac cycle. We used a nonlinear optimization algorithm to estimate Young’s modulus from the measured displacements, which we have previously described^44–46^; therefore, we will provide only a brief description of the methodology. Young’s modulus was estimated by finding the distribution that minimized the difference between the measured tissue displacements (${{\rm{U}}}_{m}$) and those computed with finite element model (${\rm{U}}$). Specifically, we minimized the following objective function:

1 $$\pi ({\rm{E}})=\tfrac{1}{2}\Vert ({\rm{U}}-{{\rm{U}}}_{m})\Vert +\tfrac{\alpha }{2}\Vert {\rm{E}}\Vert ,$$

where bold symbols represent vectors; **E** represents the Young’s modulus distribution and $\alpha$ represents the regularization parameter used to constrain the solution. Minimizing Eq. (1) with respect to Young’s modulus produces the matrix solution at the (*i* + 1) iteration as follows:

2 $${{\rm{E}}}^{i+1}={\rm{\Delta }}{{\rm{E}}}^{i}+{[J{({{\rm{E}}}^{i})}^{T}J({{\rm{E}}}^{i})+\alpha I]}^{-1}\cdot J{({{\rm{E}}}^{i})}^{T}({{\rm{U}}}^{m}-{\rm{U}}({{\rm{E}}}^{i})),$$

where *T* denotes the transpose; I is an identity matrix, $J({{\rm{E}}}^{i})$ is the sensitivity matrix at the *i* ^th^ iteration, and ${{\rm{\Delta }}E}^{i}$ is a vector of Young’s modulus updates. This inverse reconstruction method was implemented in the Python 3.14 programming language, which was compiled on a 16-core Intel Xeon Server that was operating at 2.93 GHz under the Centos 5.6 (64-bit) operating system. All reconstructions were performed using a homogeneous trial solution (E = 20 kPa). At each iteration, we used the finite element method to compute the displacement (axial and lateral) at a given Young’s modulus distribution. We solved the finite element problem by assuming the tissue under investigation exhibited linear isotropic mechanical behavior, and that it was nearly incompressible (Poisson’s ratio = 0.495). A uniform pressure (131 mm Hg) was assumed on the inner lumen and on the outer boundary we used the displacements measured by tracking to imposed Dirichlet boundary conditions.

### Morphometry

We used a cocktail of ketamine and xylazine (130 mg/kg and 9 mg/kg, i.p.) to anesthetize mice right after ultrasound imaging at 2 weeks post-surgery. All animals were perfusion fixed; the carotid arteries were harvested, processed, embedded in paraffin, and cross-sections were prepared as before^28^. Serial cross-sections were stained with hematoxylin and eosin (DAKO) and morphometry analyzed by MCID image software (Imaging Research Inc, St. Catharines, Ontario Canada) as described^28^. We measured the carotid lumen, intima, media, and adventitia areas at 200 *μ*m intervals from the carotid bifurcation and calculated compartment volumes for 2,000 *μ*m of the carotid length. It was not possible to measure the intimal layer in non-ligated arteries. Custom software using the MATLAB (The Mathworks Inc., Natick, MA, USA) programming environment was used to generate 3D-volume rendered images from the averaged compartment area over the long axis of the artery as described previously^34^. Carotid artery compartments were color-coded: black = lumen, yellow = intima, red = media, and green = adventitia. We manually removed a quarter of the area to create an open-ended solid surface of each of the vessel layers.

### Statistical analyses

We report our findings as means ± SEM. We used JMP12.0.0 software (SAS Institute Inc., Cary, NC, USA) for statistical analyses. In our estimations for group size, we have set the Alpha level at 0.05. Based on our previously published data on carotid IMT volume in FVB mice^30^, the smallest sample size that results in 95% power to detect changes of 185 × 10^6^ *μ*m^3^ with a standard deviation of 33 is n = 4. Differences between two experimental groups were examined using the Student’s *t* test. A one-way analysis of variance (ANOVA) test was applied for four experimental groups followed by post hoc comparisons (Student’s *t* test). The level of p < 0.05 was considered as significant.

### Data availability

All data generated or analyzed during this study are included in this article and Supplementary Information files. No large datasets were generated or analyzed during the current study.

## Electronic supplementary material

Supplementary Information
