# Supplementary material for: Model-based vascular elastography improves the detection of flow-induced carotid artery remodeling in mice
Source: Sci Rep. 2017 Sep 21;7:12081. doi: 10.1038/s41598-017-12321-7 (PMC5608712; doi:10.1038/s41598-017-12321-7)
Supplement: Supplementary file 1 — Supplementary Information [file 41598_2017_12321_MOESM1_ESM.pdf]

Supplementary information

Title: Model-based vascular elastography improves the detection of flow-induced carotid artery remodeling in mice

Short title: Carotid elastography in mice

Authors: Vyacheslav A. Korshunov[1,2,*], Hexuan Wang[3], Rifat Ahmed[3], Deanne M. Mickelsen[1], Qian Zhou[1], Chen Yan[1,*], and Marvin M. Doyley[3,*]

[1]Aab Cardiovascular Research Institute, Department of Medicine, and [2]Biomedical Genetics, University of Rochester School of Medicine and Dentistry, Rochester, NY USA; [3]Department of Electrical & Computer Engineering, University of Rochester and Hajim School of Engineering & Applied Sciences, Rochester, NY USA

*To whom correspondence should be addressed:
Vyacheslav "Slava" A. Korshunov, PhD
University of Rochester School of Medicine and Dentistry
Aab Cardiovascular Research Institute
601 Elmwood Ave, Box CVRI
Rochester, NY 14642
Phone: 585 276 7705
Fax: 585 276 1530
E-mail: Slava_Korshunov@URMC.rochester.edu

Co-corresponding author
Chen Yan, PhD
University of Rochester School of Medicine and Dentistry
Aab Cardiovascular Research Institute
601 Elmwood Ave, Box CVRI
Rochester, NY 14642
Phone: 585 276 7704
Fax: 585 276 1530
E-mail: Chen_Yan@URMC.rochester.edu

Co-corresponding author
Marvin M. Doyley, PhD
University of Rochester
Hajim School of Engineering and Applied Sciences
333 Hopeman Engineering Building, Box 270126
Rochester NY 14627
Tel: 585 275 3774
Fax: 585 273 4919
E-mail: m.doyley@rochester.edu

Table S1. Left carotid ligation procedure had no effect on cardiac function in FVB mice over 2 weeks time-course.

Group	1 week		2 weeks	
**Parameter**	**Sham, n=5**	**Ligated, n=5**	**Sham, n=5**	**Ligated, n=6**
Heart rate, beats/min	566 ± 13	559 ± 9	580 ± 9	570 ± 11
Systolic diameter, mm	1.88 ± 0.08	1.93 ± 0.11	1.84 ± 0.06	1.83 ± 0.09
Diastolic diameter, mm	3.26 ± 0.13	3.34 ± 0.11	3.32 ± 0.08	3.24 ± 0.13
Systolic volume, μL	11 ± 1	12 ± 2	10 ± 1	10 ± 1
Diastolic volume, μL	44 ± 4	46 ± 4	45 ± 3	42 ± 3
Stroke volume, μL	32 ± 3	34 ± 3	34 ± 2	32 ± 2
Ejection fraction, %	75 ± 1	74 ± 3	77 ± 1	76 ± 2
Fractional shortening, %	42 ± 1	42 ± 3	44 ± 1	44 ± 1
Cardiac output, mL/min	19 ± 2	19 ± 2	20 ± 1	18 ± 1
LV mass, mg	67 ± 5	69 ± 6	72 ± 6	63 ± 7
LV mass corrected, mg	54 ± 4	55 ± 5	58 ± 5	50 ± 5

LV, left ventricular. Parameters are shown as mean±SEM. n, number per group.

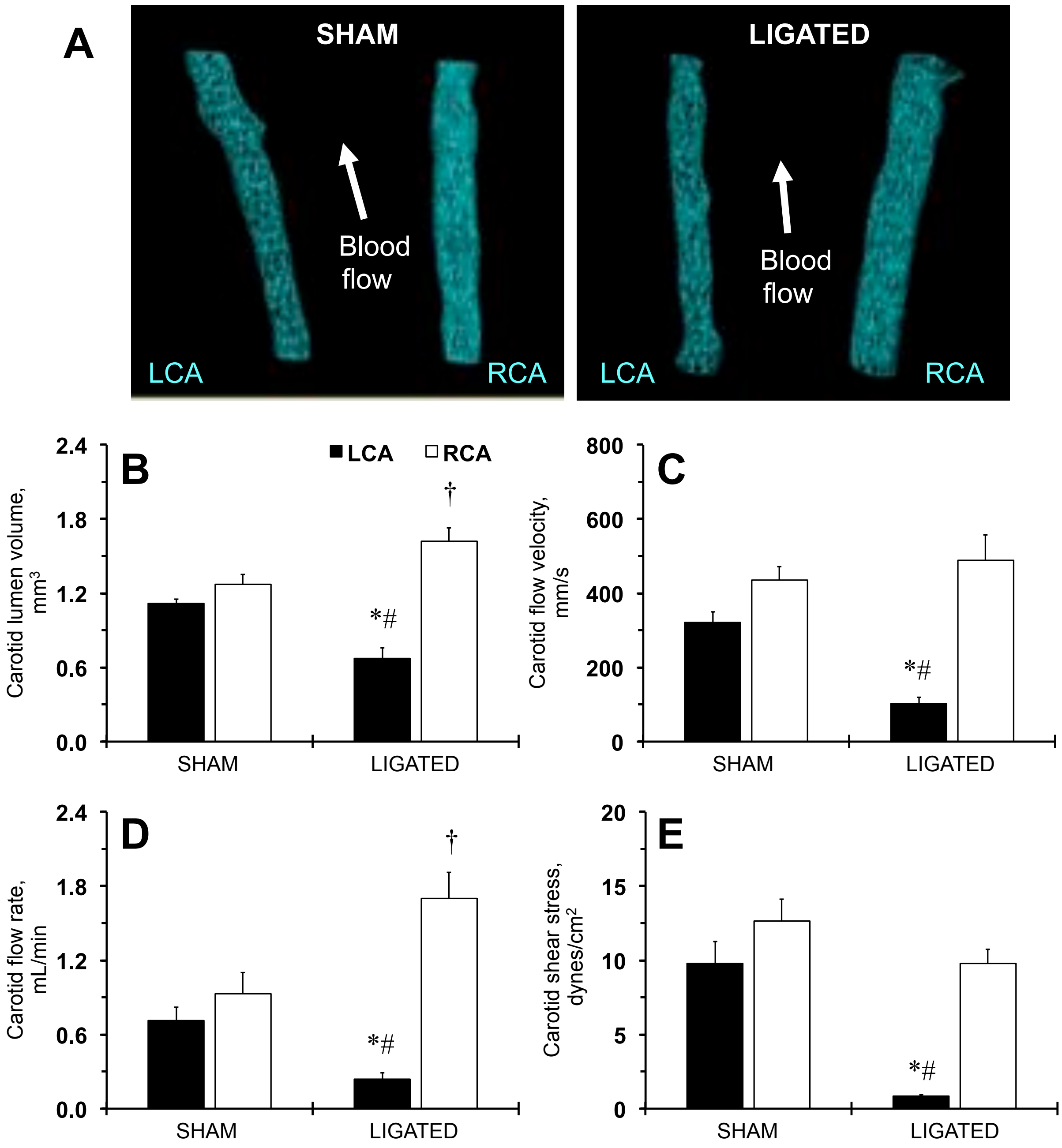

Figure S1. Hemodynamic changes in carotid arteries determined by ultrasound in FVB mice one week after ligation. A. Representative 3D-images of the lumen of the sham-operated (SHAM) or ligated (LIGATED) mice. LCA, left carotid artery. RCA, right carotid artery. Arrows show direction of the blood flow. **B.** Carotid lumen volume, mm^3. **C.** Carotid blood flow velocity rate, mm/s. **D.** Carotid blood flow volume rate, mL/min. **E.** Carotid shear stress, $dynes/cm^2$. Black bars are LCA. Open bars – RCA. SHAM and LIGATED mice are shown on X-axis. Values are mean±SEM. *, $p<0.05$ vs. LCA, SHAM. †, $p<0.05$ vs. RCA, SHAM. #, $p<0.05$ vs. RCA, LIGATED. n=5-6 per group

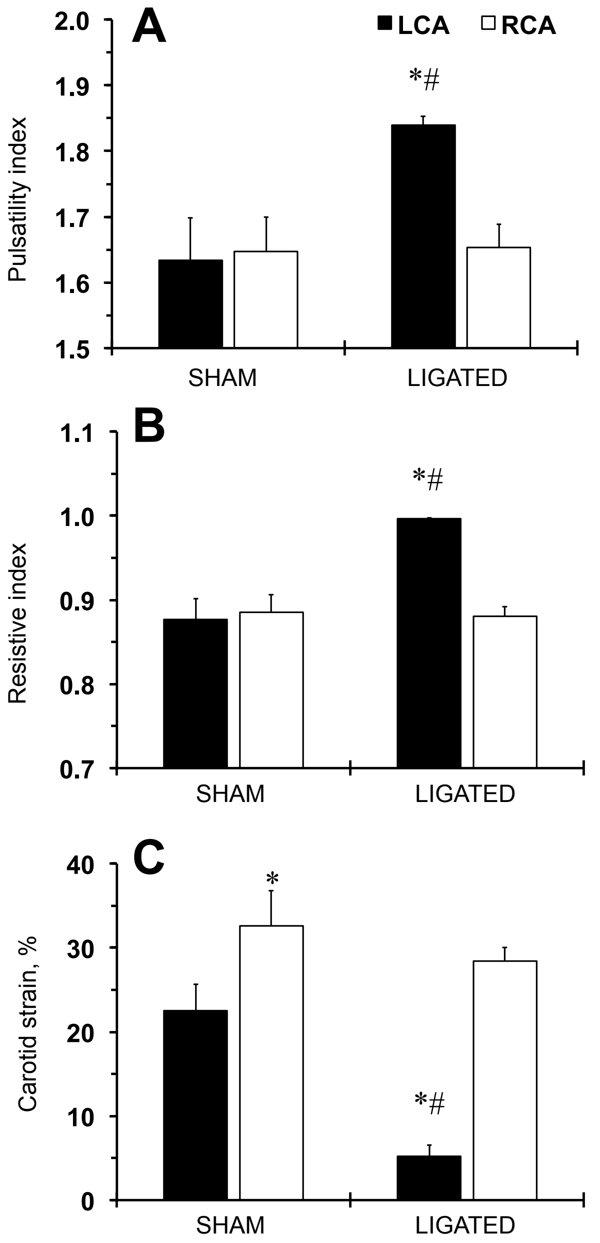

Figure S2. Ultrasound profiles of the carotid artery wall in FVB mice one week after ligation. A. Carotid artery pulsatility index. **B.** Carotid artery resistive index. **C.** Carotid artery strength, %. Black bars are left carotid artery (LCA). Open bars – right carotid artery (RCA). SHAM and LIGATED mice are shown on X-axis. Values are mean±SEM. *, $p<0.05$ vs. LCA, SHAM. #, $p<0.05$ vs. RCA, LIGATED. n=5-6 per group

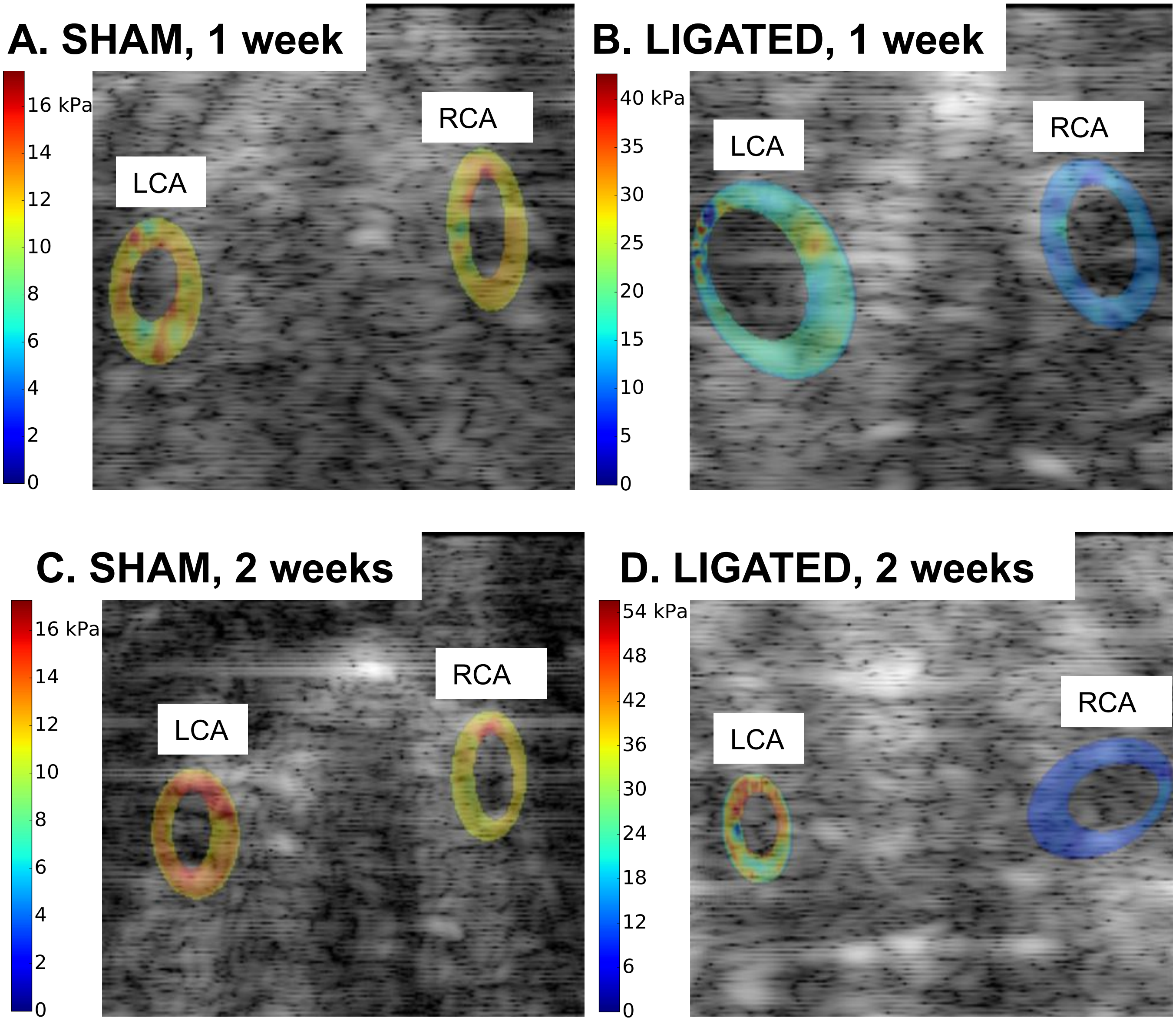

Figure S3. Elastography profiles of the carotid arteries in FVB mice after ligation. Computed sonograms of the carotid arteries from the same animal at 1 week (**A**) or 2 weeks (**C**) after sham operation. Computed sonograms of the carotid arteries from the same animal at 1 week (**B**) or 2 weeks (**D**) after ligation. LCA, left carotid artery. RCA, right carotid artery. The Young's modulus is color-coded: lowest (zero) is set to blue color; highest value (16 or 40 or 54 kPa) is in red

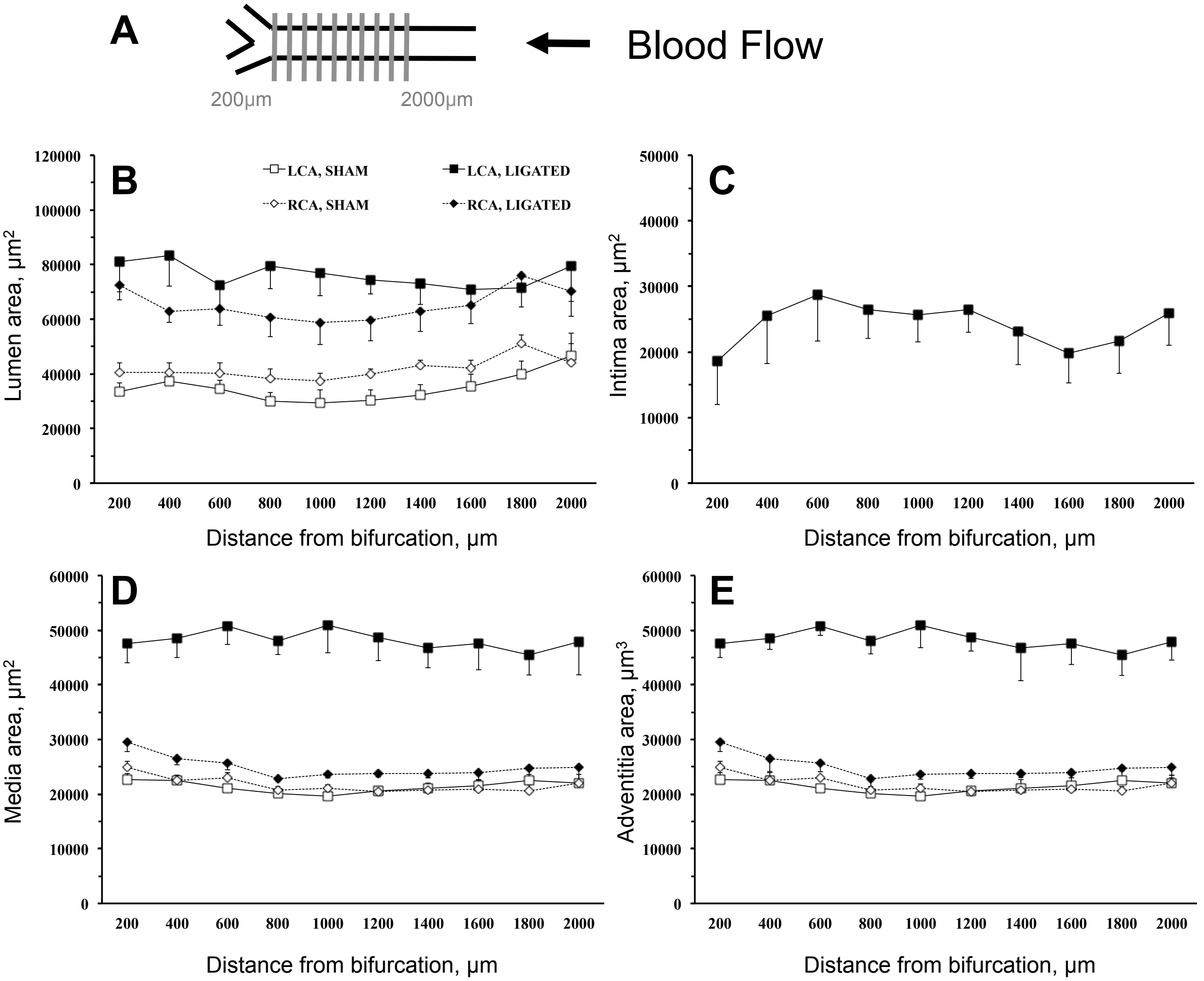

Figure S4. The carotid component areas are plotted versus the length of the carotid arteries cross-sections from FVB mice with or without ligation. A. A cartoon showing carotid artery at the bifurcation point. Series of cross-sections marked with grey perpendicular lines (every 200 µm) are covering 2,000 µm carotid artery length. Arrow shows direction of blood flow. **B.** Lumen area distribution. **C.** Intima area distribution. It was not possible to measure the intimal layer in non-ligated arteries. **D.** Media area distribution. **E.** Adventitia area distribution. Black lines and open squares are left carotid artery (LCA) from SHAM-operated mice. Interrupted lines and open rhombs are right carotid artery (RCA) from SHAM-operated mice. Black lines and black squares are LCA from LIGATED mice. Interrupted lines and black rhombs are RCA from LIGATED mice. X-axis shows distance from carotid bifurcation, µm. Values are mean±SEM. n=5-6 per group

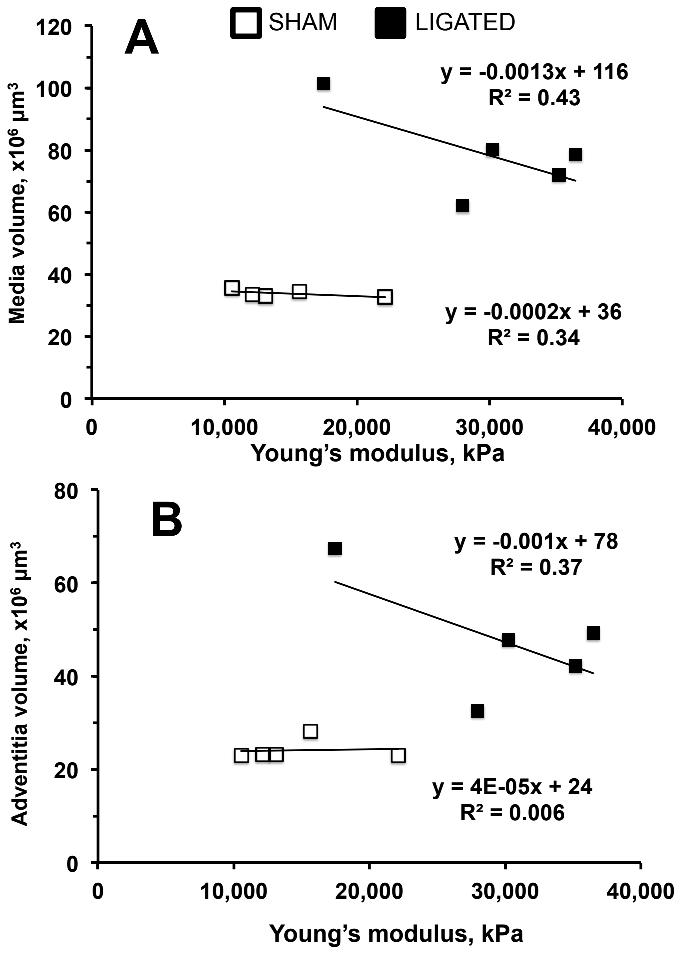

Figure S5. Relationships between the Young's modulus and the left carotid remodeling in FVB mice after 2 weeks after ligation. Open squares are sham-operated animals (SHAM). Black squares are ligated mice (LIGATED). Media volume (**A**) and Adventitia volume (**B**) negatively correlate with the Young's modulus after ligation. No correlation was noted in shams. n=5 per group
